# Validity of questionnaire-based assessment of sedentary behaviour and physical activity in a population-based cohort of older men; comparisons with objectively measured physical activity data

**DOI:** 10.1186/s12966-016-0338-1

**Published:** 2016-02-04

**Authors:** Barbara J. Jefferis, Claudio Sartini, Sarah Ash, Lucy T. Lennon, S. Goya Wannamethee, Peter H. Whincup

**Affiliations:** UCL Department of Primary Care & Population Health, UCL Medical School, Rowland Hill Street, London, NW3 2PF UK; UCL Physical Activity Research Group, University College London, London, UK; Population Health Research Institute, St George’s University of London, Cranmer Terrace, London, SW17 0RE UK

**Keywords:** Older adults, Sedentary behaviour, Physical activity, Questionnaire, Accelerometer, Cohort study

## Abstract

**Background:**

Older adults are the most inactive age group and self-reporting of activities may be complicated by age-related reductions in structured activities and misclassification or recall biases. We investigate the validity of simple questionnaires about sedentary behaviour (SB), (including the widely used proxy television (TV) viewing), and physical activity (PA) in comparison with objective measures.

**Methods:**

Community dwelling men aged 71–93 years, from a UK population-based cohort wore a GT3X accelerometer over the right hip for 7 days and self-completed a questionnaire including information about SB (TV, reading, computer use and car use) and PA (leisure and sporting domains).

**Results:**

1566/3137 surviving men (mean age 79 years) attended. 1377 ambulatory men provided questionnaire and accelerometer data. Questionnaires under-estimated mean daily sedentary time; 317 minutes total SB (TV, computer use, reading or driving), 176 minutes (TV) vs 619 minutes (objectively measured). Correlations between objective measures and self-reports were 0.18 (total SB) and 0.17 (TV), both *P* < 0.001. Objective SB levels were similar across the lowest three quartiles of self-reported SB but raised in the highest quartile. Correlations between steps/day or moderate to vigorous PA with self-reported total PA were both 0.49, *P* < 0.001 and measured PA levels were progressively higher at higher levels of self-reported PA.

**Conclusions:**

Among older men, simple SB questions performed poorly for identifying total SB time, although simple PA questions were associated with a graded increase with objectively measured PA. Future studies of health effects of SB in older men would benefit from objective measures of SB.

**Electronic supplementary material:**

The online version of this article (doi:10.1186/s12966-016-0338-1) contains supplementary material, which is available to authorized users.

## Background

Both physical activity (PA) and sedentary behaviour (SB) are core determinants of health and are associated with mortality and CVD risk in prospective cohort studies [1, 2]. UK guidelines include recommendations about both PA and SB [3]. At older ages levels of PA are lowest and SB are highest [4] and burdens of PA preventable disease and disability rise steeply with age [5]. Accurate assessment of PA and SB is therefore essential in this age group, yet there is little data about validity of both PA and SB in the oldest old, aged up to 90 years. Until recently all studies relating PA and SB to morbidity and mortality used self-reports (with television (TV) viewing the most commonly used proxy for SB) [6]. TV viewing is consistently associated with elevated risks of mortality, CVD and diabetes events [7]. Yet some studies reporting detrimental association between self-reported TV time and CVD risk markers, fail to find associations with accelerometer measured SB [8]. This may be because TV viewing accounts for only a small part of SB and does not reflect other domains (leisure, occupational and transport) [9, 10]. Furthermore, there are concerns about questionnaire-based assessment of both PA and SB in older people, in whom misclassification bias and recall bias (which could be exacerbated by memory loss) [11] are particularly likely and who report difficulty in answering questions about typical SB [12]. Moreover, this age-group undertakes fewer sporting and structured exercise and more light activities (including functional tasks such as walking for transport, household tasks, caring and gardening [13]), which may be harder to recall, or not be included in questionnaires designed for younger age groups. Sensor technology permits objective measurements of PA and SB in population-based studies, providing estimates of time spent at different intensities of activity. In order to better understand the health effects of SB and to design effective interventions to change patterns of SB in older adults, it is important to assess the validity of commonly used instruments to measure SB in large scale studies of community-dwelling older adults. Likewise, it is important to understand the validity of existing PA questionnaires which have been used to generate estimates of PA, from which the dose–response curves between PA and clinical outcomes are estimated

This paper therefore aims first to investigate how the most widely used measure of SB (TV viewing) and a more comprehensive SB score made up of common sedentary activities (TV viewing, computer use, reading and car driving time) are related to accelerometer-measured SB and different intensities of activities in the oldest old. Second, it aims to evaluate whether the established British Regional Heart Study (BRHS) PA questionnaire which has been used extensively to investigate health effects of PA [14–16], can capture the “low active” older adults who have greatest potential to benefit from activity interventions, and differentiate physical activity levels across “high active” groups. Third, it aims to investigate the properties of a single question about recreational PA which could be useful in screening for low active older individuals in time or resource-poor settings. Additionally, it investigates associations between questionnaire and objective measures of activity in older adults (content validation) and associations with FEV_1_ and heart rate as markers of fitness (construct validation).

## Methods

### Measures

#### Sample

The British Regional Heart Study is an on-going prospective, population-based cohort study following up 7735 men recruited from primary care centres in 24 British towns in 1978–80 when aged 40–59 years [17]. In 2010–2012, 3137 survivors were invited to a clinic reassessment. The National Research Ethics Service (NRES) Committee London provided ethical approval. Participants provided informed written consent to the investigation. FEV_1_ was measured using a Vitalograph Compact II Spirometer, with the subject standing. A nurse demonstrated how to blow into the mouthpiece and then the participant completed three tests (blows). The highest of three consecutive test readings was selected using American Thoracic Society criteria [18] : if the best test variation was >5%, a further test was recorded. FEV_1_ values were standardised to height squared. Men also had a resting 12-lead electrocardiogram (Burdick Atria 6100 ECG Machine) recorded, during which heart rate was measured. Men lay on a bed and rested for at least 5 minutes prior to measurement. Sensitivity analyses of Heart Rate data excluded (i) men with atrial fibrillation or tachycardia diagnosed on ECG using Minnesota coding guidelines, and (ii) men who reported taking beta blockers, which are associated with lower heart rate. Men completed a standard study questionnaire [19, 20] about PA and SB and were fitted with an accelerometer to objectively measure PA and SB.

#### Accelerometer data

Men wore a GT3x accelerometer (Actigraph, Pensacola, Florida) over the right hip for 7 days, during waking hours, removing it for bathing or swimming. Accelerometer data were processed using standard methods as previously described [21]. Valid wear days were defined as ≥600 minutes wear time, and participants with ≥3 valid days were included in analyses, a conventional requirement to estimate usual PA level [22–24]. The number of minutes per day spent in PA of different intensity levels was categorised using standard count-based intensity threshold values of counts per minute developed for older adults [25]: <100 for SB (<1.5 MET),100-1040 for light activity (1.5-3 MET) and > =1040 for MVPA,(> = 3 MET). The time spent in bouts of MVPA was labelled as MVPA1+: which is the total number of individual minutes in MVPA (irrespective of whether they occurred consecutively or not), and also as MVPA10+ which is the number of minutes spent in bouts lasting 10 consecutive minutes or more (without a break).

#### Questionnaire data

Men completed a questionnaire including questions about how many hours per week they spent (i) watching TV, videos or DVDs (ii) reading (iii) using a computer (iv) driving or sitting in a car. A total SB score was derived by summing time spent in the four activities. One question about self-rated recreational and domestic activity was asked “Compared to a man who spends two hours on most days on activities such as walking, gardening, household chores, DIY (do it yourself) projects, how physically active would you consider yourself?” (much more active/ more active/ similar/ less active /much less active). Men also completed the standard BRHS physical activity questionnaire (used since 1978); usual PA is self-reported under the headings of regular walking or cycling, recreational activity, and sporting (vigorous) activity. Regular walking and cycling relate to daily journeys. Recreational activity included gardening, pleasure walking, and do-it yourself jobs. Sporting activity included running, golf, swimming, tennis, sailing and digging. A PA score (validated in relation to heart rate and FEV_1_ [19, 20]) was derived for each man. Scores were assigned for each type of activity and duration on the basis of the intensity and energy demands of the activities reported based on Minnesota intensity codes [26]. Men were categorized into six groups based on their total score. The total score for each man is not a measure of total time spent doing PA but is a relative measure of how much PA has been carried out [20].

### Statistical methods

Quartiles of the number of hours per week of (i) total SB (sum of watching TV, reading, using a computer and driving or sitting in a car) and (ii) watching TV were calculated. The standard six category BRHS PA score was calculated for all men [20]. Spearman’s correlations between total minutes/day recorded as SB by accelerometer and (i) total sedentary hours (sum of watching TV, reading, using a computer and driving or sitting in a car) and (ii) hours watching TV were calculated. Bland Altman plots compared the self-reported total SB to accelerometer measured SB, plotting the difference between the two measures (in the same metric, minutes/day) against the average of the two measures, differences and limits of agreement were calculated. Plots were repeated for TV viewing time. Neither the questionnaire nor accelerometer are gold standard measures of SB, so these plots give evidence of concurrent rather than criterion validity. Bland Altman plots were not calculated for PA measures as the questionnaire gives ranks rather than absolute PA levels and cannot therefore be compared to accelerometer PA measures on the same scale.

Within each category of self-reported TV watching (4 categories), total sedentary time (4 categories), self-reported total activity (6 categories) and self-rated recreational and domestic activity (5 categories), mean and 95%CI of objective measures of PA and SB were calculated. The objective accelerometer measures were mean daily: (i) counts per minute (CPM) (ii) steps (iii) minutes spent in SB (iv) sedentary 60+ (SB in bouts lasting ≥ 60 minutes) (v) light PA (vi) MVPA 1+ (in bouts lasting ≥ 1 minute) (vii) MVPA10+ (in bouts lasting ≥10 minutes).

Random effects linear regression models estimated associations between quartiles of self-reported TV time and each of the accelerometer measures, accounting for clustering, with accelerometer measurement (range 3–8 days) at level 1 and person at level 2. Models were adjusted for measurement related factors; age, day order (first, second, third etc. day of wear), season, accelerometer wear time (minutes/day) and town of residence. Adjusted mean values for each objective measure of PA or SB were calculated for each self-reported SB or PA measure. Complete case analysis was used. Statistical analyses were run in R version 2.15.3 and Stata version 13 [27, 28].

## Results

Among 3137 men invited, 1655 attended (52.8 %), of whom 1455 (46.4 %) provided questionnaire and adequate accelerometer data (≥600 minutes wear time on 3–7 days) (Table 1). Analyses were restricted to 1377 independently mobile, community-dwelling men, aged on average 79 years (range 71–93), with a BMI of 27.1kg/m^2^, 22.6 % of whom had pre-existing CVD. Comparing characteristics of men who did participate in the accelerometer survey to those who did not, participants were more active at previous time points (59 % vs 46 % were at least moderately active at a survey 10 years previously), younger (mean age 78.5 years vs 80.1years), and less likely to smoke (7.2% vs 12.9 %) compared to men who did not participate.

1377 men met the criteria above and also had complete PA questionnaire data, of which 1257 also had data on HR and FEV_1_. 1112 men had complete data on SB score (men with missing data both about participating in an SB activity and hours per week in that activity were not included in the SB score, Table 1). Among the 1112 men, the average total SB was 317 minutes/day, comprising 176 minutes watching TV, 65 minutes reading, 42 minutes driving and 35 minutes using a computer. Over 90 % of men watched TV, read, drove or sat in a car but only 51.8% used a computer. 13% of men were moderately vigorously active and 9 % of men vigorously active according to the habitual PA score (Table 2). On average each day men registered 187 accelerometer counts per minute, took 4769 steps, and spent 619 minutes in SB (122 minutes in bouts lasting > =1 hour), 200 minutes in light activity and 39 minutes in MVPA. On average only 9 minutes/day were spent in bouts lasting ≥10 minutes (Table 3), reflecting the fact that many men did not sustain a single 10 minute bout of MVPA per day.

**Table 1:** Flow chart for men invited to accelerometer study at clinic visit

	N	%
Participants invited	3137	100
Agreed to participate	1655	52.8
Actigraph returned	1566	49.9
Actigraph missing/lost, n=53(1.7 %)		
Actigraph faulty, n=6 (0.2 %)		
Actigraph returned unworn, n=30 (1.0 %)		
Actigraph with a valid week	1528	48.7
With a valid questionnaire	1455	46.4
Not in residential home or in a wheelchair	1455	46.4
With PA score self-reported	1377	43.9
a) and FEV_1_ + Heart rate	1257	40.0
b) and sedentary behaviour score self-reported	1112	35.4

**Table 2:** Health, demographic, physical activity and sedentary behaviour characteristics (n = 1377 men), mean (SD) or % (n)

Men with PA score self-reported and accelerometer data^a^, n	1377
Region, % (n)	
South	36.2 (499)
Midlands	14.9 (205)
North	39.0 (538)
Scotland	9.8 (135)
Age at baseline (years), mean (SD)	78.5 (4.6)
BMI (Kg/ m^2^), mean (SD)	27.1 (3.8)
Health status	
Pre-existing CVD, % (n)	22.6 (345)
ECG Heart Rate^b^	
Heart Rate, bpm, mean (SD)	64.8 (11.9)
Men without atrial fibrillation and tachycardias, % (n)	88.4 (1111)
Men who did not use beta-blockers, % (n)	71.3 (953)
Spirometry^b^	
FEV_1_, L, mean (SD)	2.5 (0.6)
Men with between test variation of <3%, % (n)^b,c^	68.4 (858)
Men performing Lung Function test without problems, % (n)	97.5 (1226)
Physical activity score self-reported	
Inactive (0–2), % (n)	17 (228)
Occasional (3–5), % (n)	23 (321)
Light (6–8), % (n)	23 (312)
Moderate (9–12), % (n)	16 (216)
Moderately Vigorous (13–20), % (n)	13 (174)
Vigorous (> = 21), % (n)	9 (126)
Sedentary behaviour self-reported^d^	
Watching TV/video/DVDs, minutes/day, mean (SD)	176 (111)
Total SB score: watching TV/video/DVDs, reading, using computer, driving (or sitting in) a car, minutes/day, mean (SD)	317 (146)
Men who do not report any TV/video/DVD viewing, % (n)	1.4(16)
Men who do not report any reading, % (n)	9.3 (103)
Men who do not report any PC use, % (n)	48.2 (536)
Men who do not report any driving or sitting in a car, % (n)	8.6 (96)
Objectively measured physical activity	
Accelerometer number of valid days per week, mean (SD)	6.7 (0.8)
Accelerometer wear time (minutes/day), mean (SD)	854 (92)
Accelerometer wear season, % (n)	
Winter (November-February)	31.2 (477)
Spring (March-May)	21.8 (333)
Summer (June-August)	23.4 (358)
Autumn (September-October)	23.6 (360)
Counts per minute, mean (95 % CI)^e^	183 (178,189)

^a^BRHS men who met the inclusion criteria and have a self-reported PA score and a valid week of objectively measured PA

^b^BRHS men who met the inclusion criteria and have a self-reported PA score, a valid week of objectively measured PA, FEV_1_ and heart rate, n = 1257

^c^Men with non-missing observations for test variation (at least two spirometer blows), n = 1254

^d^BRHS men who met the inclusion criteria and have a self-reported PA score, a valid week of objectively measured PA, and self-reported sedentary behaviour score, n = 1112

^e^means adjusted for wear time, day order, season and region using random effects model

### Validity of self-reported sedentary behaviour: TV viewing, computer use, sitting in a car, reading

**Fig. 1:**
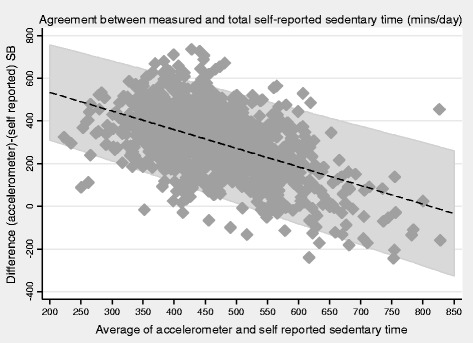
Bland Altman plot for association between self-reported total SB with objectively measured SB in n=1112 men.

**Fig. 2:**
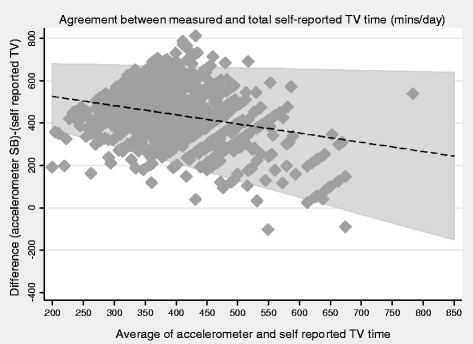
Bland Altman plot for association between self-reported SB (TV watching) with objectively measured SB in n=1112 me

Both self-reported total SB and TV viewing time were weakly positively correlated with accelerometer SB time (Spearman’s r = 0.18 and 0.17 respectively, both *P* < 0.001). Bland Altman plots showed that accelerometers recorded higher SB time than either self-report measure (Figs. 1 and 2); 300 (95 % CI 291,309) minutes/day (limits of agreement −6 to 607) more compared to self-reported total SB time and 440 (95 % CI 433,447) minutes/day more (limits of agreement 193 to 687) compared to self-reported TV viewing. For both total SB time and TV time, the discrepancy between accelerometer and self-report TV time was greater (with participants reporting much less SB time than was measured) in the participants who were least sedentary. In sensitivity analyses, no evidence was found that the mean differences varied by social class (manual vs non manual), presence of pre-existing CHD or stroke and presence of mobility limitations However, among men aged <80 years and > =80 respectively, accelerometers recorded 285(95 % CI 274,295) more minutes per day and 335(95 % CI 318, 352) more minutes per day for self-reported total SB time and equivalently 431(95 % CI 423,440) and 460(95 % CI 446,473) more minutes per day for self-reported TV viewing, suggesting that error in self-reported SB as particularly marked among the over 80s. Descriptive data for the total self-reported SB and PA levels stratified by age group are presented in Additional file 1: Table S1.

Self-reported total SB and TV viewing time were strongly correlated (Spearman’s r = 0.78, *P* < 0.001), so patterns of associations of each self-report measure with accelerometer measured SB were similar (Table 3). In both cases, men reporting highest SB levels (top quartile) had markedly higher measured SB than the rest, whereas measured SB was very similar across the lowest three quartiles of self-reported SB (Table 2). An inverse pattern was observed for the PA measures (CPM, steps, high light activity and MVPA). As noted above, men self-reported substantially less SB time than registered on accelerometers; data in Table 3 show that in the lowest quartile of the SB score, self-reported total SB score accounted for about 25 % of measured daily SB time, compared to 80 % in the top quartile of the SB score. In the lowest quartile of TV viewing, TV time accounted for about 10% of measured SB whereas it rose to 50 % in the men in the highest quartile.

### Validity of self-reported physical activity

Correlations between the self-reported PA score and accelerometer measured CPM, steps, light and MVPA were 0.49, 0.49, 0.33 and 0.49 respectively, all *P* < 0.001. Self-reported PA levels were associated with higher CPM, step count, light and MVPA and lower SB in a graded, stepwise manner (Table 4). There was a larger incremental difference in step count, CPM and high light activity between “inactive” and “occasional” groups than for increases in groups at higher activity levels. The gradient in activity level was stronger for step count and total MVPA than for low light activity. There was a plateau across the three highest categories of the PA scores for values of low light PA and bouts of SB lasting ≥ one hour. Overall 15 % of men achieved 150 minutes/week in bouts of ≥10 minutes, rising from 0.4 % in the “inactive” to 25.4 % in the “vigorously active”.

**Table 3:** Associations Between Self-Reported SB with Objectively Measured PA and SB, n = 1112 Men^a^

			Quartiles of self-reported TV watching							
	Q1 (n = 321, 28.9 %)		Q2 (n = 342, 30.8 %)		Q3 (n = 202, 18.2 %)		Q4 (n = 247, 22.2 %)		Total (n = 1112, 100 %)	
	Mean	SD or 95 % CI	Mean	SD or 95 % CI	Mean	SD or 95 % CI	Mean	SD or 95 % CI	Mean	SD or 95 % CI
Self Reports TV watching, minutes/day^b^	62	30	147	23	207	25	335	96	176	111
PA score (ranking, 0–5)	2.3	1.5	2.4	1.5	2.4	1.6	1.7	1.4	2.2	1.5
Accelerometer data^c^ CPM	204	192,217	192	181,204	185	171,199	157	145,168	187	180,193
SB (<100 CPM)	606	597,614	614	606,622	616	606,626	647	638,657	619	615,624
SB in bouts ≥60 minutes^e^	112	102,122	117	109,126	110	100,121	144	131,157	120	115,126
Light PA (100–1040 CPM)	206	200,213	205	199,211	201	193,208	182	174,190	200	196,203
MVPA 1+ (>1040 CPM)^f^	44	40,47	41	37,44	38	34,43	32	28,35	39	37,41
MVPA10 + (bouts ≥10 minutes)^g^	11	10,13	9	8,11	9	7,11	7	6,9	9	9,10
			Quartiles of self-reported total SB score							
	Q1 (n = 282, 25.4 %)		Q2 (n = 284, 25.5 %)		Q3 (n = 276, 24.8 %)		Q4 (n = 270, 24.3 %)		Total (n = 1112, 100 %)	
	Mean	SD or 95 % CI	Mean	SD or 95 % CI	Mean	SD or 95 % CI	Mean	SD or 95 % CI	Mean	SD or 95 % CI
Self Reports Total SB score, minutes/day^d^	150	51	264	24	351	26	513	105	317	146
PA score (ranking, 0–5)	2.3	1.5	2.3	1.5	2.4	1.6	1.9	1.5	2.2	1.5
Accelerometer data^c^ CPM	202	188,215	190	178,202	191	178,203	162	151,174	187	180,193
SB (<100 CPM)	599	589,609	614	606,622	622	613,631	644	635,653	619	615,624
SB in bouts ≥60 minutes^e^	110	100,121	117	107,127	115	106,125	140	128,152	120	115,126
Light PA (100–1040 CPM)	208	201,216	207	200,213	203	197,210	179	172,186	200	196,203
MVPA 1+ (>1040 CPM)^f^	43	39,47	40	36,43	40	36,43	33	30,37	39	37,41
MVPA10 + (bouts ≥10 minutes)^g^	11	9,12	9	8,11	10	8,12	8	7,10	9	9,10

PA physical activity; SB sedentary behaviour; CPM counts per minute; MVPA moderate or vigorous physical activity

^a^BRHS men who met the inclusion criteria: had a self-reported PA score and a valid week (≥3 days of ≥600 minutes) of accelerometer data

^b^Mean (SD) total time watching television, min/day

^c^Coefficients are mean (95 % CI) minutes per day spent in each level of activity, adjusted for wear time, day order, season and region using random effects model.

^d^Mean (SD) total SB score (watching television + using a computer + reading + sitting in a car), min/day

^e^Sedentary 60+ (total number of minutes of SB in bouts lasting ≥ 60 minutes)

^f^MVPA 1+ (total number of minutes of MVPA in bouts lasting ≥ 1 minute)

^g^MVPA10+ (total number of minutes of MVPA in bouts lasting ≥10 minutes)

**Table 4:** Associations Between Self-Reported PA Score and Components of Objectively Measured PA^a^ in n = 1337 Men^b^

	Inactive 0–2 (n = 228, 17 %)		Occasional 3–5 (n = 321, 23 %)		Light 6–8 (n = 312, 23 %)		Moderate 9–12 (n = 216, 16 %)		Moderately Vigorous 13–20 (n = 174, 13 %)		Vigorous > =21 (n = 126, 9 %)		Total (n = 1377, 100 %)	
	Mean	95 % CI	Mean	95 % CI	Mean	95 % CI	Mean	95 % CI	Mean	95 % CI	Mean	95 % CI	Mean	95 % CI
CPM	94	87,102	152	143,161	197	186,209	215	202,228	231	216,246	268	248,289	183	178,189
Steps	2385	2190,2581	3945	3724,4166	5192	4919,5466	5606	5279,5932	6133	5759,6507	6809	6369,7250	4769	4630,4908
SB (<100 CPM)	671	663,680	634	627,642	611	603,619	598	589,608	598	588,608	581	567,594	620	616,624
SB in bouts >60 min.^c^	180	164,195	129	118,139	110	102,118	102	93,111	100	90,110	98	88,109	122	117,127
Low light PA, (101–759 CPM)	137	131,144	172	166,178	185	180,190	197	191,204	190	184,196	203	194,211	178	176,181
High light PA (760–1040 CPM)	10	9,11	16	15,17	20	19,21	24	22,25	23	21,24	28	25,30	19	18,20
MVPA 1+ (>1040 CPM)^d^	14	12,16	28	26,30	42	38,45	47	43,51	53	49,58	64	58,70	38	37,40
MVPA 10+ (bouts ≥10 minutes )^e^	1	1,2	6	5,7	12	10,14	12	10,14	13	10,15	15	13,18	9	9,10

^a^Coefficients are Mean (95% CI) minutes per day spent in each level of activity, adjusted for wear time, day order, season and region using a random effects model.

^b^BRHS men who met the inclusion criteria and have a self-reported PA score and a valid week of objectively measured PA

^c^Sedentary 60+ (total number of minutes of SB in bouts lasting ≥ 60 minutes)

^d^MVPA 1+ (total number of minutes of MVPA in bouts lasting ≥ 1 minute)

^e^MVPA10+ (total number of minutes of MVPA in bouts lasting ≥10 minutes)

The associations between self-reported and objectively measured PA did not vary by presence of pre-existing CVD, or by age group (interactions all p > 0.2). The only exception was the presence of interactions between age group and both CPM and total MVPA minutes (likelihood ratio test, *P* = 0.017 and *P* = 0.046 respectively). Among younger men, the PA scores were associated with higher objectively measured CPM and MVPA minutes than among older men, and this difference was greater at the higher scores for the younger men.

Total PA score was linearly and inversely related to resting heart rate and positively related to FEV_1_ (Additional file 1: Table S2).

### Validity of self-reported recreational activity

Responses to a single item question about how much recreational activity men did compared to other men their age were positively and linearly associated with mean CPM, daily steps, minutes of light PA and of MVPA1+ (Additional file 1: Table S3). Correlations between this single item PA question and accelerometer measured CPM, steps, light and MVPA were 0.46, 0.45, 0.42 and 0.43 respectively, all *P* < 0.001. The prevalence of adhering to MVPA guidelines increased though flattened out in the two most active categories. Mean daily minutes in sedentary behaviour and long sedentary bouts decreased linearly, although again there was a plateau at higher scores. Self-reported recreational activity was positively related to HR and FEV_1_ (Additional file 1: Table S2).

## Discussion

In this large sample of community dwelling older men, the validity of self-reported SB was weak (either as a score with four sedentary behaviours or just TV viewing), with correlations of <0.2. The top quartile of the self-reported SB score identified the most sedentary men, however measured SB and PA were similar across the three lower quartiles. Self-report SB scores had poor content validity and performed most poorly among the men aged over 80 years. In contrast, we found evidence for content and construct validity for both the PA score (a simple to administer self-report based on different domains of activity (habitual active transport; walking and cycling, recreational and sporting activities)) and a single item recreational PA question. Both were strongly positively associated both with objective measures of total MVPA and step counts, and inversely with long and short bouts of SB, demonstrating content validity and associated with measures of fitness (higher FEV_1_ and lower Heart Rate), demonstrating construct validity. Hence both were valid for ranking PA in older adults, although they cannot necessarily identify the actual intensity of physical activity level.

### Validity of self-reported Sedentary behaviours

As seen in other studies [29, 30], accelerometers recorded much more SB time than either the self-reported total SB score (including reading, car and computer use) or just TV score. This is partly because the scores omit some habitual SBs (such as eating, sitting talking, resting). Further there may be social desirability bias (where undesirable activities are underestimated) or recall bias (that activities are systematically forgotten). However recall bias specifically about SB seems unlikely, given that both self-reported PA scores performed very well in relation to objective measures. The four components of our SB score are reported to be among the most common individual SBs in other studies of older adults examining more individual SBs, furthermore they are among the most reliably recalled, with highest test-retest ICCs, compared to other behaviours [9, 31].

In our study, we did not observe differences in performance of the SB scores by age group, presence of CVD and mobility limitations, except that the SB self-report performed better in the under 80s, which fits with other data showing better performance of an SB score in <75 year olds [31]. Overall, our correlations between SB scores and measured SB of <0.2 fit with data from other studies of older adults investigating the validity of self-reported TV-watching compared to objective measures which report correlations of 0.23 for >60 year olds [30] and 0.22 in 65–92 year olds [32] – in the latter study all nine other single SB questions were also correlated ≤0.2 with accelerometer measured SB. A different single item question asking about the time spent sitting in an average day also had poor agreement with accelerometer measured SB (Kappa −0.0003) [33]. Given that TV and the other domains of SB we asked about accounted for a smaller proportion of the total daily SB time in the lower compared to the higher quartiles of the self-report scores, we were interested in whether self-report SB scores derived from questionnaire instruments including more domains of SB might perform better. However correlations between accelerometer measured SB and self-report SB were 0.3 for a 12-item instrument [31], 0.14 [33] and 0.12 [10] respectively for 9 and 8 item scores derived from different modifications of the SB questions on the Community Health Activities Model Program for Seniors (CHAMPS) questionnaire, and 0.3 [9] for a 7-item questionnaire designed to measure SB in older adults. A correlation of 0.35 for a 10-item instrument, was maximised to 0.46 by selecting a sub-set of 6 items however sleep was included in both of these self-report scores [32], so they are not directly comparable to other studies reported here, as sleep is specifically excluded from definitions of SB [34]. Hence the validity of self report scores for assessing total time in SB appears to be limited in older adults, although self-reported SB questionnaires may give insight into the context of behaviours. Our findings have implications for studies of self-reported SB in relation to health outcomes; the poor discrimination of the lower levels of self-reported SB time in relation to objective PA and SB may account for some of the discrepancies in the reported relations between cardio-metabolic markers and self-reported TV time compared to objectively measured SB [8, 35, 36].

### Validity of self-reported Physical activity

Our results suggested good content and construct validity for the six-point PA score: correlations between the score and step counts, MVPA and CPM were 0.5; stronger than for the SB score and than for some other PA questionnaires [37]. Also, the PA score differentiated between individuals performing across the range of PA levels, including at low activity levels. Hence the score could help identify low-active individuals who would most benefit from PA interventions. The PA score also differentiated total measured MVPA well. However the higher PA scores performed less well at identifying MVPA in bouts lasting at least 10 minutes (needed to fulfil current PA guidelines) and light physical activity; hence the questionnaire may misclassify light activity levels in some of the more active adults. The total physical activity score differentiated sedentary behaviours well: both total amount and prolonged bouts of SB declined steadily as the score increased.

Single item screening questions asking participants to rank themselves against others have been used in many large scale epidemiologic studies because they are simple to administer and have a low participant burden. They are often used to control for total amount of physical activity as a confounding variable. Our results suggested good content and construct validity of our single item question although there was a ceiling effect, so the question was better at ranking low-active than highly active individuals. This is important in older populations given that many other questionnaires are reported to perform more poorly in identifying low active adults. Our measure may be useful in population-based studies requiring a ranking of older adults’ PA levels.

### Strengths and limitations

We studied concurrent objectively measured and self-reported SB using commonly used activity questions and accelerometers in a large population based- sample of free-living community-dwelling older men recruited from across the UK. Whilst the cut point we used for defining MVPA from the accelerometer data are validated in relation to energy expenditure in older adults (8), SB defined as <100 CPM from an Actigraph accelerometer worn over the hip could include some standing time, however average CPM was <10 CPM during SB. Therefore varying the definition of SB from <100 to <50 CPM changed the total SB time recorded very little, so any biases are likely to be small. Hip-worn Actigraph-measured SB has been demonstrated to have minimal bias compared to thigh-worn Activpal measured SB and the two measures correlate r = 0.76 [38]. Accelerometer study response rates (50%) are similar or superior to other studies of older adults; in other studies valid data were obtained from 21 % [23], 43 % [22] and (in the Health Survey for England population) 37 % of women and 48 % of men over 75 years had 4 or more days with valid data [39]. Nevertheless, participants were more often younger, and had healthier behaviours than non-participants and may therefore have been more physically active and less sedentary than the general population. As this study includes only men results may not be generalizable to older women, who have different patterns of PA [21] and may have different reporting biases or recall. Indeed it has been reported that TV time is a better indicator of SB time for women than for men [40]; future studies should investigate the properties of self-reported compared to objectively measured SB in older women.

## Conclusions

Our simple PA score is useful in older adult populations for differentiating the high and low active and classifying the men across low activity levels, but may misclassify light intensity activity. The single screening question about recreational activities performed well for grading all intensities of PA and SB. Validity of self-reported SB was poor overall, especially at lower levels of SB, both for the single TV viewing question and for the combined 4-domain measure. This suggests that objective assessment of SB is likely to be more accurate than self-reported measures similar to those used in this study. As discussed above, more extended SB instruments used in other studies also related poorly to objective SB measures in older adults. Given the widespread use of TV viewing as a proxy for SB in population studies, there may be implications for accurate estimation of the health effects of SB, particularly among individuals who watch less TV. Hence there is a need for studies examining the health effects of PA and also SB to use objective assessments.

## Additional file

Additional file 1: Table S1. Physical activity and sedentary behaviour characteristics, mean (SD) or % (n), stratified by age 80 years. **Table S2.** Associations Between PA and SB Score and Heart Rate (Beats Per Minute) and FEV_1_ (Litres). **Table S3.** Associations between self-reported recreational activity score and components of objectively measured PA (n=1337 men).^1^ (DOCX 21 kb)

## Abbreviations

BMI: Body mass index
CHD: Coronary heart disease
CPM: Counts per minute
CVD: Cardiovascular disease
ECG: Electrocardiogram
FEV_1_: Forced expiratory volume in one second
HR: Heart rate
MET: Metabolic equivalent of task
MVPA: Moderate to vigorous physical activity
NRES: National research ethics service
PA: physical activity
SB: sedentary behaviour
TV: television
