# Supplementary material for: Validity of questionnaire-based assessment of sedentary behaviour and physical activity in a population-based cohort of older men; comparisons with objectively measured physical activity data
Source: Int J Behav Nutr Phys Act. 2016 Feb 4;13:14. doi: 10.1186/s12966-016-0338-1 (PMC4743260; doi:10.1186/s12966-016-0338-1)
Supplement: Additional file 1: Table S1. — Physical activity and sedentary behaviour characteristics, mean (SD) or % (n), stratified by age 80 years. Table S2. Associations Between PA and SB Score and Heart Rate (Beats Per Minute) and FEV1 (Litres). Table S3. Associations between self-reported recreational activity score and components of objectively measured PA (n=1337 men).1 (DOCX 21 kb) [file 12966_2016_338_MOESM1_ESM.docx]

**Supplementary Table 1.
Physical activity and sedentary behaviour characteristics, mean (SD) or % (n), stratified by age 80 years.**

|  | Age <80 years | Age >= 80 years | P(no difference) |
| --- | --- | --- | --- |
| **Physical activity score self-reported** |  |  | <0.001 |
| Inactive (0-2), % (n) | 12 (95) | 20 (69) |  |
| Occasional (3-5), % (n) | 22 (167) | 25 (87) |  |
| Light (6-8), % (n) | 21 (157) | 27 (93) |  |
| Moderate (9-12), % (n) | 16 (124) | 16 (55) |  |
| Moderately Vigorous (13-20), % (n) | 16 (121) | 9 (31) |  |
| Vigorous (>=21), % (n) | 13 (98) | 4 (15) |  |
| **Sedentary behaviour self-reported** ^4^ |  |  |  |
| Watching TV/video/DVDs, minutes/day, mean (SD) | 173.1 (108.0) | 179.1 (119.1) | 0.416 |
| Total SB score: watching TV/video/DVDs, reading, using a computer, driving (or sitting in) a car, minutes/day, mean (SD) | 321.4 (142.3) | 307.7 (151.7) | 0.133 |
| Men who do not report any TV/video/DVD viewing, %(n) | 1.2(9) | 2.0(7) | 0.287 |
| Men who do not report any reading, %(n) | 8.4 (64) | 11.1 (39) | 0.143 |
| Men who do not report any PC use, %(n) | 41.6 (317) | 62.6 (219) | <0.001 |
| Men who do not report any driving or sitting in a car, %(n) | 5.6 (43) | 15.1 (53) | 0.008 |

**Supplementary Table 2.
Associations Between PA and SB Score and Heart Rate (Beats Per Minute) and FEV_1_ (Litres)**

|  | **Heart rate, bpm** | | | | | **FEV_1,_ L** | | | | |
| --- | --- | --- | --- | --- | --- | --- | --- | --- | --- | --- |
|  | **All** | | **Without atrial fibrillation / tachycardia** | | **All** | | | **Non-smokers** | | |
|  | n | Mean(SD) | N | Mean(SD) | N | | Mean(SD) | n | Mean(SD) |  |
| **Self-reported PA score^1^** |  |  |  |  |  | |  |  |  |  |
| Inactive (0-2) | 192 | 66.2(13.1) | 170 | 65.2(12.4) | 192 | | 2.1(0.6) | 182 | 2.1(0.6) |  |
| Occasional (3-5) | 285 | 65.3(12.1) | 244 | 64.8(11.8) | 285 | | 2.4(0.6) | 274 | 2.4(0.6) |  |
| Light (6-8) | 291 | 64.6(11.4) | 260 | 64.2(11.3) | 291 | | 2.5(0.6) | 286 | 2.5(0.6) |  |
| Moderate (9-12) | 207 | 65.7(12.7) | 189 | 65.0(12.1) | 207 | | 2.5(0.6) | 191 | 2.6(0.6) |  |
| Mod Vigorous (13-20) | 164 | 63.8(10.8) | 148 | 63.8(10.5) | 164 | | 2.6(0.5) | 162 | 2.6(0.5) |  |
| Vigorous (>=21) | 118 | 61.4(9.8) | 100 | 59.5(8.3) | 118 | | 2.7(0.6) | 114 | 2.7(0.6) |  |
| Total | 1257 | 64.8(11.9) | 1111 | 64.1(11.5) | 1257 | | 2.5(0.6) | 1209 | 2.5(0.6) |  |
| **Self-reported recreational activity score^2^** | | |  |  |  | |  |  |  |  |
| Much less active | 89 | 65.7(11.1) | 76 | 65.8(10.6) | 89 | | 2.1(0.6) | 81 | 2.1(0.6) |  |
| Less active | 203 | 65.2(12.6) | 168 | 63.6(11.3) | 203 | | 2.3(0.6) | 199 | 2.3(0.6) |  |
| Similar | 465 | 64.7(11.4) | 416 | 64.1(11.1) | 465 | | 2.5(0.6) | 446 | 2.5(0.6) |  |
| More active | 257 | 63.9(11.3) | 235 | 63.4(11) | 257 | | 2.6(0.6) | 245 | 2.6(0.6) |  |
| Much more active | 98 | 63.3(11.6) | 86 | 63.2(11) | 98 | | 2.6(0.6) | 98 | 2.6(0.6) |  |
| Total | 1112 | 64.6(11.6) | 981 | 63.9(11) | 1112 | | 2.5(0.6) | 1069 | 2.5(0.6) |  |

1 Leisure time PA score including sporting activities and regular walking and cycling
2 Compared with a man who spends four hours on most weekends on activities such as walking, gardening, household chores, DIY projects, how physically active would you consider yourself?

**Supplementary Table 3
Associations between self-reported recreational activity score and components of objectively measured PA (n=1337 men)^1^.**

|  | Much less active (n=124, 11.2%) | | Less active (n=272, 24.5%) | | Similar  (n=563, 50.6%) | | More active (n=309, 27.8%) | | Much more active (n=109, 9.8%) | | Total  (n=1377, 100%) | |
| --- | --- | --- | --- | --- | --- | --- | --- | --- | --- | --- | --- | --- |
|  | Mean | 95% CI | Mean | 95% CI | Mean | 95% CI | Mean | 95% CI | Mean | 95% CI | Mean | 95% CI |
| CPM | 84 | 76,93 | 128 | 120,137 | 193 | 185,201 | 231 | 218,243 | 248 | 225,270 | 183 | 178,189 |
| Steps | 2075 | 1838,2311 | 3404 | 3186,3623 | 5042 | 4848,5237 | 5992 | 5685,6299 | 6292 | 5781,6803 | 4767 | 4629,4905 |
| SB (<100 CPM) | 676 | 665,687 | 651 | 643,658 | 617 | 611,623 | 592 | 584,600 | 577 | 562,592 | 620 | 616,625 |
| SB in bouts >60 minutes | 189 | 168,210 | 147 | 135,158 | 109 | 103,116 | 109 | 100,118 | 90 | 78,103 | 122 | 117,127 |
| Low light, (101-759 CPM) | 131 | 122,141 | 154 | 148,159 | 183 | 179,187 | 197 | 192,202 | 217 | 207,227 | 178 | 176,181 |
| High light (760-1040 CPM) | 9 | 7,10 | 13 | 12,14 | 20 | 19,21 | 24 | 23,26 | 27 | 25,30 | 19 | 18,20 |
| MVPA 1+ (>1040 CPM) | 12 | 9,14 | 23 | 20,25 | 41 | 38,43 | 52 | 48,56 | 55 | 49,62 | 38 | 37,40 |
| MVPA 10+ (bouts ≥10 minutes ) | 1 | 0,1 | 5 | 4,6 | 10 | 9,12 | 14 | 12,16 | 13 | 10,16 | 9 | 9,10 |

^1^ Coefficients are Mean (95% CI) minutes per day spent in each level of activity, adjusted for wear time, day order, season and region using random effects model
